# Comparative analysis of different methods for protein quantification in donated human milk

**DOI:** 10.3389/fped.2024.1436885

**Published:** 2024-10-01

**Authors:** Elisabet Navarro-Tapia, Ana Herranz Barbero, Maribel Marquina, Cristina Borràs-Novell, Vanessa Pleguezuelos, Rafael Vila-Candel, Óscar García-Algar, Vicente Andreu-Fernández

**Affiliations:** ^1^ Faculty of Health Sciences, Valencian International University (VIU), Valencia, Spain; ^2^ Grup de Recerca Infancia i Entorn (GRIE), Institut de Recerca Agustí Pi i Sunyer (IDIBAPS), Barcelona, Spain; ^3^ Neonatology Department, BCNatal-Centre de Medicina Maternofetal i Neonatologia de Barcelona, Hospital Clínic, Universitat de Barcelona, Barcelona, Spain; ^4^ Biosanitary Research Institute, Valencian International University (VIU), Valencia, Spain; ^5^ Catalan Department of Health, Banc de Sang i Teixits (BST), Barcelona, Spain; ^6^ La Ribera Health Department, Midwifery Primary Health, Alzira, Spain

**Keywords:** protein quantification, infrared, Bradford assay, ultrasonic, MIRIS, human milk

## Abstract

**Background:**

Human milk is the best option for feeding newborns, especially premature infants. In the absence of breast milk, milk from a human milk bank can be a suitable alternative. However, the nutritional content of human milk may be insufficient to meet these high requirements and milk fortification is needed. To facilitate the implementation of simpler and faster analyzers in neonatal healthcare facilities, this study focuses on the concordance analysis of two different analyzers, one based on mid-infrared and the other on ultrasound, in comparison to the Bradford method for determining protein concentration in human milk.

**Methods:**

Mature milk samples from donor mothers were collected and pasteurized at the Human Milk Bank of Barcelona and protein quantification was performed using mid-infrared (MIRIS-HMA), ultrasound (MilkoScope Julie27), and the classical Bradford reference methods. The intraclass correlation coefficient (ICC) with 95% confidence interval and Bland–Altman plots were used to assess the agreement between methods.

**Results:**

The mean protein concentration of 142 milk samples calculated using MIRIS-HMA, MilkoScope, and the Bradford assay were 1.38, 1.15, and 1.19 g/100 ml, respectively. The ICC was 0.70 for MIRIS-HMA vs. Bradford and 0.37 for MilkoScope vs. Bradford.

**Conclusion:**

MIRIS-HMA obtained a better agreement with the Bradford technique and is a promising method for developing new devices based on MIR transmission spectroscopy principles. This study confirms how MIRIS-HMA can be used to accurately calculate the protein concentration of human milk.

## Introduction

Human milk (HM) is the best option to nourish newborns. Breastfeeding is an essential physiological process that provides multiple health benefits, such as protection against the development of allergies, improvement in long-term neurodevelopmental outcomes, protection against necrotizing enterocolitis, or reduction in cardiovascular disease risk factors in childhood (1–3). In addition, a meta-analysis showed that children aged 6–11 months who were not breastfed had an increased risk of mortality (4). The American Academy of Pediatrics (AAP) has stated that human milk is the optimal overall food. In fact, a decade later, the Academy has updated its guidelines on breastfeeding and supports continued breastfeeding for 2 years or more, “as mutually desired by mother and child” (5). In case a mother's own milk is not available, WHO and UNICEF recommend banked human milk as the best alternative, especially for preterm and other vulnerable infants (6). The increase in the number of human milk banks (HMBs) provides a nutritional feeding alternative for preterm infants in approximately 66 countries (7). Human milk contains many bioactive proteins involved in the modulation of the immune system and defense against pathogens, probiotic effects, inhibition of growth of pathogens, enzyme activities, enhancement of nutrient absorption, and growth stimulation (8, 9). Milk delivered to HMBs should be pasteurized to inactivate bacterial and viral agents that can compromise the health of newborns, especially premature infants. Low-temperature long-time pasteurization (LTLT), known as Holder pasteurization (HoP), is the reference method used worldwide. HoP consists of heating the milk to 62.5°C for a long duration (30 min) and quick cooling below 4°C (10, 11). The pasteurization treatment affects the biological and nutritional properties of the product and some soluble vitamins, particularly vitamin C, as well as certain proteins with immunological and anti-infective activity, such as lactoferrin and immunoglobulins (7).

Neonatal nutritional requirements differ according to gestational age and individual physiological developmental characteristics. Very preterm infants (VPI) have high nutrient requirements, approximately 115–140 kcal/kg/day, with 3.5–4 g/kg protein per day to promote adequate growth and development (12). Despite all other clear benefits, the nutrient content of human milk is insufficient to meet these high demands, and fortification is required to increase the concentration of protein, calcium, and phosphorus (13). However, standard fortification does not consider the variability in the macronutrient content of human milk or the differing requirements of infants. As a result, using a standardized approach may lead to protein deficits in preterm infants (14). Standard fortification typically provides the recommended energy intake but cannot provide sufficient protein for many very low birth weight infants (15). Therefore, it is important to analyze the nutritional content in milk to adjust the protein levels based on each infant's metabolic response (known as individualized fortification). In addition, the European Milk Bank Association (EMBA) encourages the use of individualized fortification to optimize nutrient intake by considering each infant's protein requirements, thus avoiding protein undernutrition and overnutrition (16). Furthermore, a recent systematic review showed that individualized fortification led to improved growth velocities in weight, length, and head circumference at the end of the intervention compared to the standard method in preterm infants (17).

Due to the variability in the macronutrient content of breast milk, individualized fortification is preceded by an analysis of the maternal or donated milk protein concentration (18). Several methods to measure the concentration of proteins in HM have been described, such as fast protein liquid chromatography, polyacrylamide gel electrophoresis, ion-exchange chromatography, enzyme activity measurement, or different types of immunoassays. Most of these techniques have unknown sensitivities and require sample pretreatment or long incubation periods (19–21). Moreover, their use requires a wet lab and high starting volumes (5–10 ml), which are not practical for daily use in the neonatal intensive care unit (NICU). Human milk analyzers (HMAs) based on mid-infrared (MIRIS-HMA) allow us to easily and quickly obtain the macronutrient profile using a small amount of milk (a sample of 3 ml), with results available in 60 s. The MIRIS-HMA, for example, was developed specifically for HM and has been recognized by the Food and Drug Administration (FDA) as safe and effective for the measurement of fat, carbohydrate, and protein in breast milk (22).

Unlike MIRIS-HMA, MilkoScope Julie Z7 is an ultrasonic method based on high-frequency ultrasound radiation and has been calibrated for human milk macronutrient content (23). However, it requires a larger volume of milk (5 ml). There are very few studies on the use or intended use of ultrasound in HM (23, 24), and there is a lack of publications comparing it with reference methods.

Few studies have been conducted to compare the methods for measuring protein concentration in HM and the results are variable and conflicting regarding their performance, accuracy, and reproducibility (25–28). To provide new and clearer information in this area, we designed a prospective study to determine protein levels using two rapid methods (MIRIS-HMA, based on mid-infrared transmission spectroscopy, and MilkoScope Julie Z7, based on ultrasound) with the Bradford test, a routine, inexpensive, and reference biochemical method for protein measurement in research laboratories. This colorimetric technique is easy to use, requires a small sample, and allows obtaining fast and accurate results compared to the Kjeldahl reference method, which is time-consuming and requires specific instrumentation (26, 29). In addition, the Bradford test has been shown to have the highest correlation with the Kjeldahl protein analysis and the lowest variability compared to other protein colorimetric assays in HM samples (30). Therefore, the objective of our study was to compare these two techniques for rapid protein measurement in HM with respect to the classical Bradford technique and to evaluate the agreement between the data sets. The results of the study can be used as a reference when choosing a protein quantification method to use in the NICU.

## Materials and methods

### Research design and samples

The protein content of pasteurized mature human milk samples was analyzed using three different techniques: MIR (using MIRIS-HMA developed by Miris, Sweden); ultrasound (using MilkoScope Julie Z7 developed by Scope Electric, Bulgaria); and a classical reference method (the Bradford test). Donations were made at the HMB of Banc de Sang i Teixits (a public company of the Health Department of Catalonia, Spain) after signing the informed consent form. Only those mothers who met the health requirements to be milk donors were included after a blood test. Mothers who smoked, consumed alcoholic beverages, had chronic or infectious diseases, took regular medication, or used drugs or other intoxicants were excluded.

This study was approved by the Ethics Committee of the Parc de Salut of the Hospital del Mar of Barcelona (reference no. PSMar 2011/4287/I) following the Declaration of Helsinki.

Donor mothers were provided with a breast pump, sterile equipment for the collection of samples, and a collection protocol. The areola, nipple, and hands were washed before milk extraction. Milk was collected directly into containers provided by the milk bank or sterilized plastic bags. The containers were filled to no more than 75% of their capacity. The milk came from a single feed and was taken from one breast. Sampling was not restricted to a specific time window as no circadian variation in total protein has been observed (31). The samples came from a single extraction and were stored at −20°C in a sterile container immediately after collection and collected by HMB staff. At the HMB, the HM was ultrasonically homogenized (75% amplitude; 1.5 s/ml milk) using an ultrasound processor (model VCX 130PB; Sonics and Materials, Newtown, CT, USA) to avoid agglutination of fats and pasteurized using the Holder method. From each pasteurized sample, three aliquots were reserved for protein determination by three different techniques: 5 ml for MIR, 12 ml for ultrasound, and 5 ml for the Bradford assay.

### Devices and assays used

The MIRIS-HMA (Miris, Sweden) is based on the MIR technique. Fundamental vibrations in the MIR spectrum are associated with different chemical groups that are directly related to fat, protein, and lactose. The specific chemical groups are measured at different wavelengths (proteins are detected at a wavelength of 6.5 µm), so the amount of energy absorbed at the specific wavelengths of the macronutrients is proportional to their concentration (32). In the case of proteins, MIR-HMA is calibrated according to the Kjeldahl method of analyzing the total nitrogen content and, according to the FDA, is effective for measuring fat, carbohydrate, and proteins in breast milk (22).

The MilkoScope Julie Z7 (Scope Electric, Bulgaria) is based on ultrasound or acoustic spectroscopy techniques and has three calibration channels that can be set for animal, soya, or human milk. Like MIRIS-HMA, this device is also calibrated according to the Kjeldahl method of analyzing protein content in human milk. MilkoScope calculates protein content by detecting the differences in the attenuation and transmission of milk constituents. Acoustic spectroscopy has a lower cost than infrared spectroscopy, and the accuracy of this technique is ±0.01% (33). However, although it can predict the concentration of macronutrients in HM, its use in this matrix has been limited to a few studies (23, 33).

The Bradford (Bio-Rad Laboratories, CA, USA) reference laboratory method is a traditional and accurate method for protein determination in which Coomassie Brilliant Blue G-250 binds to protein, causing a shift in the absorption maximum of the dye from 365 to 595 nm (34).

### Protein quantification

Before analyzing the true protein content of the pasteurized samples, the HM samples were thawed in a thermostatic bath at 40°C and homogenized using an ultrasonic processor (model VCX 130PB; Sonics and Materials, Newtown, CT, USA; 75% amplitude and 1.5 s/ml milk). The ultrasound technique provides more accurate homogenization, which is essential for a correct nutritional analysis and for obtaining a higher absolute concentration of macronutrients (35).

A protein analysis was carried out using two different devices calibrated for human milk analysis: MIRIS-HMA and MilkoScope Julie Z7, according to the manufacturers' instructions. Sample volumes of 3 ml were used for the MIRIS-HMA analysis and 10 ml for the MilkoScope Julie Z7, and sample readings were taken 1–2 min after analysis using the instrument. The Bradford assay was performed to measure and compare the protein concentration between instruments and to assess the agreement between them. The Bradford assay was performed using a commercially available protein reagent (Bio-Rad Laboratories, Richmond, CA, USA) and bovine serum albumin fraction V (Sigma-Aldrich, Steinheim, Germany) to generate a standard curve for the calculated protein concentration. The use of this protein standard was in line with previous studies using this reference method for HM (27, 36). Similarly, a recent comparative study has shown that protein content measured using the Bradford test in pasteurized milk samples with different levels of fat and total solids contents is in agreement with the Kjeldahl method (26). In addition, it has a lower variability compared to other protein colorimetric assays in human milk samples (30). The sample volume used was 1 ml and the entire process took approximately 30 min. To minimize the human factor as much as possible, all sample treatments before analysis were performed by one researcher. Samples were measured after thawing and stored in the fridge to avoid freeze/thaw cycles that could compromise sample stability.

### Data collection

Written informed consent was obtained from all participants. Information about study participants remained confidential and was managed according to the “good clinical practice” requirements. The milk protein analysis data were collected in Excel and each sample was coded to ensure confidentiality.

### Data analysis

A statistical analysis was performed using SPSS® version 20.0 (IBM Corp., Armonk, NY, USA). Correlation and concordance between MIRIS-HMA, MilkoScope Julie Z7, and the Bradford assay (used a reference technique) were assessed via intraclass correlation coefficient (ICC). The ICC was interpreted according to the Munro classification system: little or no correlation for values in the range of 0–0.25; low correlation for values of 0.26–0.49; moderate correlation for values of 0.50–0.69; high correlation for values of 0.70–0.89; and very high correlation for values in the range of 0.90–1.00 (37). A Bland–Altman plot was used to analyze the agreement between the different assays and the established reference laboratory method (Bradford assay). The Bland–Altman plot is a method used to compare two measurement techniques and to assess the agreement between two sets of data. The plot provides a visual representation of the difference between two measurements on the *y*-axis and the mean of the two measurements on the *x*-axis. If the difference between the measurements (bias) is close to zero and the 95% limits of agreement (LOA) are within the clinically acceptable range, the measurements are considered to be in good agreement. Duplicate measurements were not performed because the Bland–Altman analysis is not an appropriate method for comparing repeated measurements as it was initially designed for two sets of measurements taken on a single occasion. The use of means would result in an underestimation of the variance of the differences from the original measurement (38).

## Results

### Protein determination in samples

Protein determination using MIRIS-HMA, MilkoScope, and the Bradford test was performed on 142 donated human milk samples from 88 donor mothers (from their 5th to 12th weeks of lactation, mean 8.12 weeks). The mean ± SD age of the mothers was 33.45 ± 4.2 years and the gestational age of infants was between 36 and 42 weeks. Overall protein concentration was in the range of 0.45–2.20 g/100 ml (mean 1.24 g/100 ml). Protein values measured using MIRIS-HMA were in the range of 0.50–2.2 g/100 ml (mean ± SD 1.38 ± 0.22 g/100 ml), while MilkoScope (ultrasound method) provided a concentration in the range of 0.98–1.52 g/100 ml (mean ± SD 1.15 ± 0.09 g/100 ml). Concentrations measured by the Bradford method were in the range of 0.45–2.19 g/100 ml (mean ± SD 1.19 ± 0.27 g/100 ml).

### Bland–Altman analysis

**Figure 1:**
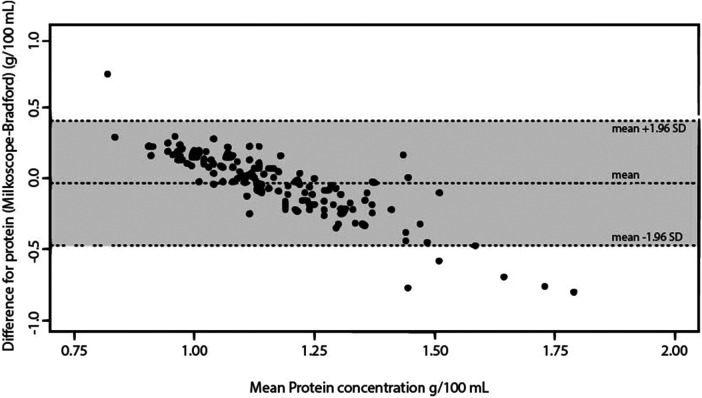
Bland–Altman plot for protein analysis showing the differences between the MilkoScope and Bradford techniques. The middle line indicates the average difference between the two methods, whereas the outer lines represent ±1.96 SD or the 95% limit of agreement.

**Figure 2:**
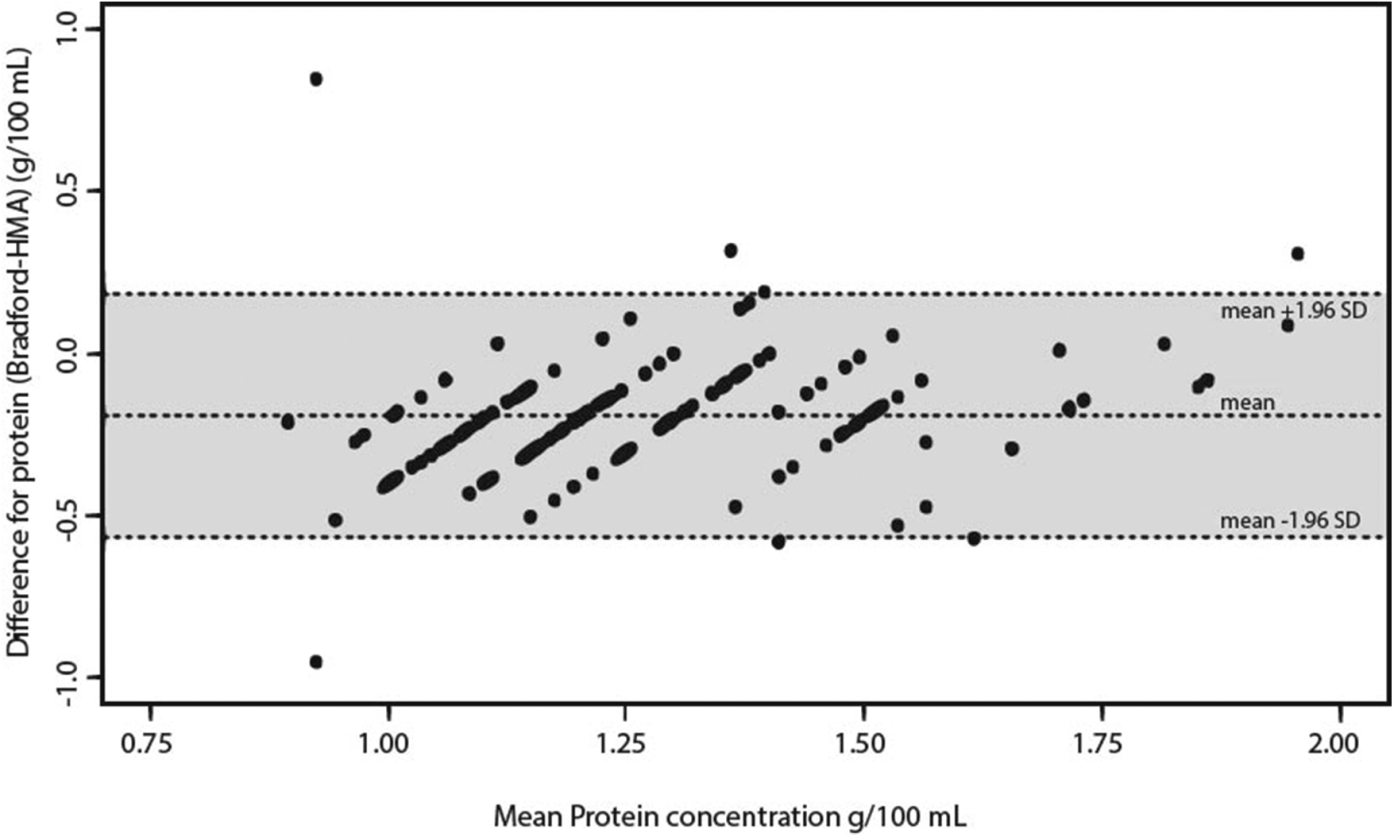
Bland–Altman plot for protein analysis showing the differences between the Bradford and MIRIS-HMA techniques. The middle line indicates the average difference between the two methods, whereas the outer lines represent ±1.96 SD or the 95% limit of agreement.

**Figure 3:**
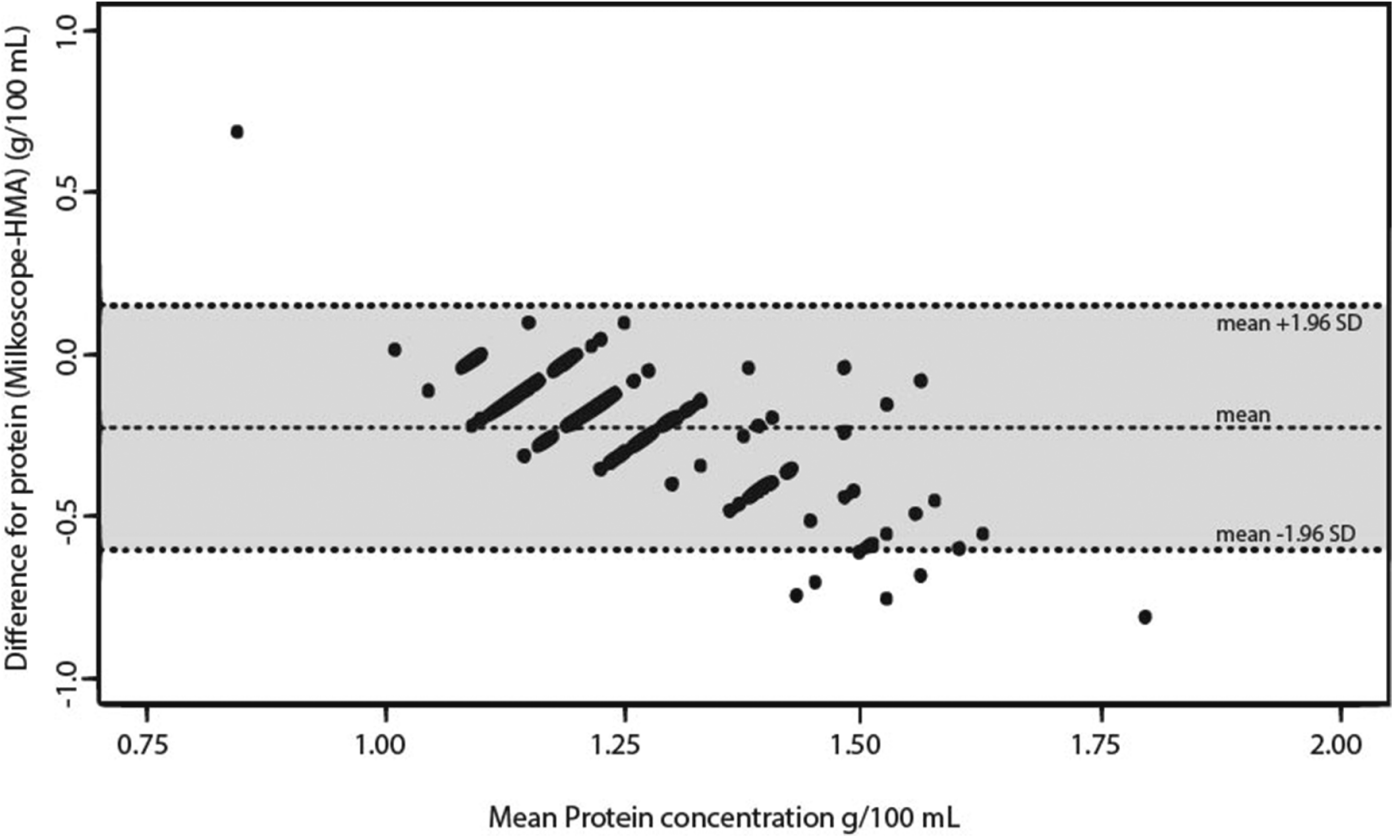
Bland–Altman plot for protein analysis showing the differences between the MilkoScope and MIRIS-HMA techniques. The middle line indicates the average difference between the two methods, whereas the outer lines represent ±1.96 SD or the 95% limit of agreement.

The ICC measures the proportion of variability in the new method that is due to real differences between subjects. It is calculated as the ratio of between-subject variance to the total variance. Any remaining variability is considered a systematic difference (39). There was moderate agreement between the three tools (ICC 0.54). Bradford vs. MIRIS-HMA yielded a substantial agreement (ICC 0.70) compared with MilkoScope vs. Bradford (ICC 0.37) and MilkoScope vs. MIRIS-HMA (ICC 0.40). Figures 1–3 show a scatter plot of the differences plotted against the average protein concentrations determined: MilkoScope vs. Bradford [mean ± SD −0.03 ± 1.96 (0.40, −0.47)], Bradford vs. MIRIS-HMA [mean ± SD −0.19 ± 1.96 (0.18, −0.56)], and MilkoScope vs. MIRIS-HMA [mean ± SD −0.22 ± 1.96 (0.15, −0.6)]. The Bland–Altman plot revealed that the scatter around the bias line was greater at higher average protein levels (Figure 1). A concordance analysis of Bradford vs MIRIS-HMA using the Bland–Altman plot showed that most individual samples from all groups were within the LOA, compared to MilkoScope vs. MIRIS-HMA or MilkoScope vs. Bradford.

### Comparison between methods

A comparison of the two methods is shown in Table 1, where we have added observations on “possible bias” and “intraclass correlation coefficient vs. Bradford assay” from this study as well as information provided by the manufacturers. All raw data about protein concentration can be found in Supplementary Table S1.

**Table 1:** Comparative table between the two analyzers used in the study.

	MilkoScope Julie Z7	MIRIS-HMA
Technology	Ultrasounds	Filtered mild-infrared spectroscopy
Volume (ml)	5–10	1–3
Sample reading (min)	1–5	1
Measurable protein range (g/100 ml)	Not provided	0.6–2.4
Accuracy (%)	0.02^a^	±15^c^
Repeatability (%)	±0.02^a^	≤3^d^
Intraclass correlation coefficient vs. Bradford assay	0.37^b^	0.70^b^
Cost	Lower	Higher
Weight (kg)	1.5–2 kg	3.8 kg
Calibration	Automatic	Manual
FDA approved for HM measurements	No	Yes
Possible bias	Risk of underestimation at high protein concentrations^b^	Not observed^b^

^a^ Data obtained from (40).

^b^ Data obtained from this study.

^c^ Data obtained from (41).

^d^ Data obtained from (15).

## Discussion

To support the implementation of rapid analyzers in clinical practice, we evaluated the difference in protein concentration in HM using different analyzers based on MIR and ultrasound. Our results showed that MIRIS-HMA obtained a better agreement using the Bradford technique and an underestimation of the MilkoScope at high protein concentrations compared to Bradford and MIRIS-HMA.

The ICC, as a Bland–Altman analysis, shows a substantial concordance between Bradford and MIRIS-HMA (ICC 0.70). The Bland–Altman plot for the comparison between Bradford and MIRIS-HMA (Figure 2) showed that most of the samples analyzed were within ±1.96 SD of zero. This finding is consistent with a study performed to validate MIRIS-HMA with a modified Bradford assay (42) in which the authors reported a significantly higher protein concentration using MIRIS-HMA compared with the Bradford method (mean difference 0.19–0.2 g/100 ml in both cases). In contrast to the results of the above-mentioned study, our study did not find that high protein concentrations tended to show greater differences between the results obtained by the Bradford and HMA methodologies. The Bland–Altman plot indicated that most of the individual samples from all groups were within the LOA. In our study, a maximum of 5% of the concentrations obtained (7 of the 142) were outside the range, demonstrating the agreement between the two quantitative methodologies. Billard et al. (36) also found an increase in protein with MIRIS-HMA compared to the Bradford method (0.3 g/100 ml). However, this mean concentration decreased after applying an adjustment in the calibration of the device, resulting in mean values of 1.2 g/100 ml. The Bland–Altman plot also showed a homogeneous distribution of the measurements, with only one outlier outside of the LOA. Surprisingly, Silvestre et al. obtained the same protein values as us using the Bradford technique (1.19 g/100 ml) (27). However, the values predicted by MIRIS-HMA (in contrast to what has been seen so far) were significantly lower (0.59 g/100 ml) and the linear correlation between them was low. The mean protein concentration in mature HM is approximately 1.0–1.2 g/100 ml (43) and the author attributed these unexpected results to the use of non-certified human milk samples for the internal calibration of the analyzer, which may have influenced the results. Currently, this system already includes this internal calibration control kit and a MIRIS control for zeroing the analyzer. Once again, the need to adjust and calibrate MIRIS-HMA before quantifying proteins is emphasized. Indeed, it is important to note that a recent international multicenter study found that the accuracy of MIRIS-HMA measurements can be improved by establishing individual correction algorithms and implementing good clinical laboratory practice (44). Furthermore, the authors concluded that recalibration of the device is necessary whenever there is a software update or the device is replaced with a new one (avoiding the use of the correction algorithm from the old one).

Despite its good reproducibility and high recovery rate, the use of milk analyzers based on ultrasounds is very limited in HMBs, so there is very little literature on the subject (23). Recently, Ruan et al. demonstrated, as we have now done, a significant difference between this technique and that of MIR (45). Although the authors used different ultrasonic (HMA 3000) and MIR analyzers (HMIR-05), they obtained higher protein levels using the MIR method (mean difference = 0.98 g/100 ml). These results support the hypothesis that the two techniques give significantly different results in terms of protein concentration, regardless of the brand used. However, the authors adjusted the results of the ultrasound method using machine learning and the mathematical model generated allowed a good fit between both methods and comparable results.

We observed a low correlation between ultrasound and MIR (ICC 0.40) and ultrasound and Bradford (0.37). Although only six measurements were outside the LOA, we observed a negative slope (Figures 1, 3), indicative of a possible systematic bias in one of the measurement techniques. That is, in our study, the ultrasound method tended to give lower protein readings when the samples had a high amount of protein. Ultrasound velocity is very sensitive to viscosity and the intermolecular interactions in the sample, so an increase in protein concentration could cause this difference between methods (46). Heterogeneity in the composition of milk (fats, proteins, carbohydrates) may alter the measurement of the device by converting the ultrasonic energy into heat, resulting in an attenuation of the ultrasound (47). Considering that the amount of protein in colostrum is significantly higher than in mature milk (50%–60% higher, depending on whether it is term or preterm human milk) (48), further studies focusing on the use of ultrasound in colostrum to determine its feasibility in measuring protein concentration are mandatory. This is important as an underestimation of the amount of protein in the HM sample could lead to over-fortification of the milk, increasing its osmolarity. This increased osmolarity combined with a higher protein component could lead to delayed gastric emptying. In addition, the development of feeding intolerance has recently been linked to high rates of fortification (49). Therefore, daily control of the instrument and its correct calibration is crucial, with recalibration of the instrument whenever necessary. In our case, we cannot know if this underestimation of the Julie Z7 at high protein concentrations is due to a bias of the device itself or to a calibration error, so it would be convenient to apply adjusted calibration curves and calibrate the device based on the type of sample to be tested. Despite their affordable cost and ease of use, further studies are needed to determine the reliability of rapid measurements (feasible in NICUs) to determine protein concentration in high content samples such as colostrum.

Standardization of the pre-analytical phase is key for validating results, as techniques such as MIR are susceptible to heterogeneity in sample preparation (28). In this study, we chose to homogenize the samples using ultrasound, regardless of the protein measurement method used. This reduces inter-sample variability and ensures optimal homogenization and sample preparation when measuring macronutrients (35).

In this study, we have seen how the use of the MilkoScope Julie Z7 can lead to an underestimation of the protein concentration in HM, while the use of MIRIS-HMA shows good agreement with classical protein measurement techniques, such as the Bradford assay. Our conclusions on the difficulty of measuring protein with the MilkoScope Julie Z7 are already mentioned in a previous review, which indicates that the fat can be measured more reliably than carbohydrates and protein (50). Although in this study we recommend the use of MIRIS-HMA for the measurement of proteins in breast milk and its use to aid in the nutritional management of newborns has been approved by the FDA (22), it is also important to note that there are cases in which its use is not recommended. Certain substances, such as citalopram, sertraline, ampicillin, vancomycin, clindamycin, cephalexin, and pseudoephedrine, may cause interference with MIRIS-HMA measurement results (51). Therefore, measuring proteins in milk from mothers taking these drugs should be avoided.

One of the strengths of our study is that we have used the same type of milk (mature), as its content is relatively stable between 2 and 12 weeks of lactation and this helps avoid inter-sample variability (48). Likewise, we have not distinguished between preterm milk and term milk because, although there are differences of up to 35% in the amount of protein in the milk in the first few days, there are no differences in protein concentration at 10–12 weeks (48). Another strength is the use of unique human samples to perform the analyses, which allow the performance of realistic and clinical conditions compared to the use of a reduced number of pooled samples observed in previous studies (36, 42, 52).

A reliable method for determining the nutrient content of HM is an important clinical tool for achieving adequate protein intake, especially for very preterm and very low birth weight infants. To process donated HM, not only should HMBs use validated and standardized methods for estimating the concentration of macronutrients, but the training of technicians in the handling and calibration of the equipment is also essential. In addition, regular quality control of the equipment should be carried out to assess its performance.

A limitation of our study is that the sample size was relatively small and that the development of mathematical adjustments would have been desirable. Although valid, it is known that certain changes in the formulae or adjustments to the equipment can produce values closer to those obtained using a reference method (53). However, we wanted to focus on the day-to-day work in neonatal facilities and test their rapid measurement devices without having to change their settings. Given the lack of validated models for these rapid techniques to date, it is essential to deepen predictive modeling and big data studies to minimize measurement errors. The choice of analyzers is another limitation; only two commercially available analyzers were used for comparison. However, the two analyzers were based on completely different principles of protein measurement (ultrasound vs. MIR). In this way, we can obtain a first approximation of the correlation between the two techniques with a classical reference technique and open the door to the development of new devices based on these technological principles.

According to the results of our study, we concluded the following: (1) MIRIS-HMA obtained a better agreement with the Bradford technique; (2) further studies are needed on the use of samples with a high protein load such as colostrum in MilkoScope, as well as the application of a proper calibration to avoid an underestimation of readings; and (3) due to the multiple steps from milk collection to protein value, it is necessary to develop validated standards and train professionals to reduce measurement errors, both in the pre-analytical and analytical phases.

## Data Availability

The original contributions presented in the study are included in the article/Supplementary Material, further inquiries can be directed to the corresponding author.
