# Supplementary material for: Comparative analysis of different methods for protein quantification in donated human milk
Source: Front Pediatr. 2024 Oct 1;12:1436885. doi: 10.3389/fped.2024.1436885 (PMC11473365; doi:10.3389/fped.2024.1436885)
Supplement: Supplementary file 1 [file Table1.docx]

Supplementary table 1. Protein concentration measurements for each of the samples used in this study.

|  | **Protein Concentration g/100 mL** | | |
| --- | --- | --- | --- |
| **ID** | **MIRIS-HMA** | **MilkoScope** | **Bradford** |
| 1 | 1.9 | 1.35 | 1.82 |
| 2 | 1.2 | 1.04 | 0.95 |
| 3 | 1.6 | 1.17 | 1.22 |
| 4 | 1.4 | 1.1 | 1.28 |
| 6 | 1.3 | 1.13 | 1.11 |
| 7 | 1.4 | 1.19 | 0.45 |
| 8 | 1.6 | 1.24 | 1.41 |
| 9 | 1.4 | 1.12 | 1.19 |
| 10 | 1.3 | 1.14 | 0.99 |
| 11 | 1.1 | 1.2 | 1.13 |
| 12 | 1.5 | 1.31 | 1.41 |
| 13 | 1.4 | 1.07 | 0.99 |
| 15 | 1.4 | 1.1 | 1.11 |
| 16 | 1.6 | 1.21 | 1.42 |
| 17 | 1.3 | 1.12 | 1.04 |
| 18 | 1.1 | 1.09 | 0.85 |
| 19 | 1.3 | 1.11 | 0.91 |
| 20 | 1.2 | 1.04 | 0.85 |
| 21 | 1.2 | 1.12 | 0.93 |
| 22 | 1.6 | 1.2 | 1.41 |
| 24 | 1.6 | 1.2 | 1.38 |
| 25 | 1.2 | 0.98 | 0.69 |
| 26 | 1.8 | 1.25 | 1.63 |
| 27 | 1.6 | 1.52 | 1.35 |
| 28 | 1.8 | 1.31 | 1.63 |
| 29 | 1.3 | 1.16 | 1 |
| 30 | 1.8 | 1.1 | 1.33 |
| 31 | 1.2 | 1.12 | 1.07 |
| 32 | 1.2 | 1.15 | 1 |
| 33 | 1.4 | 1.09 | 0.95 |
| 34 | 1.2 | 1.17 | 1.1 |
| 35 | 1.6 | 1.18 | 1.43 |
| 36 | 1.3 | 1.16 | 1.09 |
| 37 | 1.2 | 1.18 | 1.05 |
| 38 | 1.3 | 1.03 | 0.9 |
| 39 | 1.3 | 1.12 | 1.04 |
| 40 | 1.3 | 1.09 | 1.12 |
| 41 | 1.3 | 1.09 | 1.09 |
| 42 | 1.3 | 1.15 | 1.3 |
| 43 | 1.8 | 1.35 | 2.11 |
| 44 | 1.7 | 1.28 | 1.43 |
| 45 | 1.2 | 1.1 | 0.92 |
| 46 | 1.4 | 1.24 | 1.2 |
| 47 | 1.4 | 1.1 | 0.9 |
| 48 | 1.4 | 1.1 | 1.18 |
| 49 | 1.4 | 1.21 | 1.22 |
| 50 | 1.2 | 1.04 | 0.93 |
| 51 | 1.4 | 1.19 | 1.3 |
| 52 | 1.4 | 1.07 | 1.09 |
| 53 | 1.4 | 1.26 | 1.31 |
| 54 | 1.4 | 1.15 | 1.03 |
| 55 | 1.4 | 1.13 | 1.35 |
| 56 | 1.3 | 1.16 | 0.98 |
| 58 | 1.3 | 1.1 | 1.01 |
| 59 | 1.2 | 1.2 | 1.15 |
| 61 | 1.2 | 1.09 | 0.99 |
| 63 | 1.9 | 1.15 | 1.33 |
| 64 | 1.3 | 1.04 | 0.87 |
| 65 | 1.1 | 1.06 | 1.02 |
| 66 | 1.3 | 1.13 | 1.12 |
| 67 | 1.5 | 1.1 | 1.32 |
| 68 | 1.8 | 1.19 | 1.51 |
| 70 | 1.4 | 1.2 | 1.22 |
| 72 | 1.7 | 1.19 | 1.12 |
| 73 | 1.2 | 1.23 | 1 |
| 74 | 1.3 | 1.25 | 1.02 |
| 75 | 1.1 | 1.1 | 1.02 |
| 76 | 1.4 | 1.2 | 1.24 |
| 77 | 1.6 | 1.19 | 1.43 |
| 79 | 1.6 | 1.14 | 1.25 |
| 81 | 1.4 | 1.08 | 1.3 |
| 82 | 1.1 | 1.07 | 0.92 |
| 83 | 1.4 | 1.36 | 1.38 |
| 85 | 1.2 | 1.18 | 0.96 |
| 86 | 1.2 | 1.19 | 1.08 |
| 87 | 1.2 | 1.25 | 1.25 |
| 88 | 1.3 | 1.09 | 1.19 |
| 89 | 1.3 | 0.99 | 1.24 |
| 91 | 2.2 | 1.39 | 2.19 |
| 92 | 1.3 | 1.22 | 1.44 |
| 93 | 1.4 | 1.26 | 1.10 |
| 94 | 1.3 | 1.02 | 1.06 |
| 97 | 1.3 | 1.14 | 1.46 |
| 98 | 1.6 | 1.19 | 1.52 |
| 99 | 1.4 | 1.23 | 1.31 |
| 100 | 1.2 | 1.10 | 0.97 |
| 101 | 1.2 | 1.03 | 0.80 |
| 102 | 1.2 | 1.11 | 1.09 |
| 103 | 1.4 | 1.16 | 1.34 |
| 104 | 1.3 | 1.14 | 1.10 |
| 105 | 1.3 | 1.11 | 1.27 |
| 106 | 1.3 | 1.11 | 1.15 |
| 107 | 1.2 | 1.04 | 0.91 |
| 108 | 1.7 | 1.26 | 1.71 |
| 109 | 1.1 | 1.07 | 0.97 |
| 110 | 1.3 | 1.09 | 0.92 |
| 111 | 1.2 | 1.05 | 0.89 |
| 112 | 1.3 | 1.12 | 1.16 |
| 113 | 1.4 | 1.05 | 1.17 |
| 114 | 1.6 | 1.24 | 1.32 |
| 115 | 1.6 | 1.25 | 1.36 |
| 116 | 1.3 | 1.18 | 1.49 |
| 117 | 1.6 | 1.16 | 1.13 |
| 118 | 1.3 | 1.12 | 1.14 |
| 119 | 1.3 | 1.18 | 0.90 |
| 120 | 1.9 | 1.22 | 1.80 |
| 121 | 1.4 | 1.12 | 1.19 |
| 122 | 1.2 | 1.02 | 0.79 |
| 124 | 1.5 | 1.28 | 1.46 |
| 125 | 1.5 | 1.46 | 1.56 |
| 126 | 1.4 | 1.26 | 1.33 |
| 127 | 1.9 | 1.30 | 1.99 |
| 128 | 1.8 | 1.06 | 1.83 |
| 129 | 1.6 | 1.45 | 1.44 |
| 130 | 1.2 | 1.16 | 1.31 |
| 131 | 1.3 | 1.05 | 1.07 |
| 132 | 1.2 | 1.08 | 0.93 |
| 133 | 1.5 | 1.25 | 1.49 |
| 134 | 1.2 | 1.30 | 1.52 |
| 135 | 1.6 | 1.36 | 1.39 |
| 136 | 1.4 | 1.18 | 1.28 |
| 137 | 1.4 | 1.14 | 1.40 |
| 138 | 1.8 | 1.22 | 1.66 |
| 139 | 1.2 | 1.05 | 1.07 |
| 140 | 1.2 | 1.11 | 0.81 |
| 141 | 1.3 | 1.17 | 1.16 |
| 142 | 1.1 | 1.08 | 0.91 |
| 143 | 1.2 | 1.07 | 0.82 |
| 144 | 1 | 1.02 | 0.79 |
| 145 | 1.3 | 1.08 | 0.87 |
| 146 | 1.2 | 1.00 | 1.02 |
| 147 | 1.3 | 1.12 | 1.10 |
| 148 | 1.6 | 1.12 | 1.47 |
| 149 | 1.2 | 1.06 | 0.87 |
| 150 | 1.4 | 1.13 | 1.22 |
| 151 | 0.5 | 1.19 | 1.35 |
| 153 | 1.5 | 1.16 | 1.38 |
| 154 | 1.3 | 1.09 | 1.17 |
| 155 | 1.1 | 0.99 | 0.83 |
| 156 | 1.3 | 1.13 | 1.10 |
| 157 | 1.8 | 1.21 | 1.27 |
| 158 | 1.4 | 1.19 | 1.08 |
| Average | 1.38 | 1.16 | 1.19 |
| SD | 0.22 | 0.10 | 0.28 |
